# Irregularity of the Teeth

**Published:** 1846-12

**Authors:** 


*Irregularity of the Teeth.*—We have a plaster model of an upper denture and alveolar ridge, presented to us for the museum of the Baltimore College of Dental Surgery, by A. Hill, dentist of Norwalk, Conn., representing a most remarkable case of irregularity. So much are the palatine arch and alveolar border contracted, that the first molars are only about seven-eighths of an inch apart and the alveolar arch at the first bicuspids, is less than half an inch wide. The width of that part of the arch occupied by the second molars is about an inch and five-eighths, but anterior to these teeth it is very compressed, and on the median line in front, it forms an acute angle. The cuspidati are nearly an inch apart, the left lateral incisor is wanting, and the right lateral and left central are nearly in contact, while the right central is situated immediately in front of them.—*Balt. Ed.*

